# Trends in the Prevalence of Multiple Chronic Conditions in Taiwan From 2000 to 2010: A Population-Based Study

**DOI:** 10.5888/pcd11.140205

**Published:** 2014-10-23

**Authors:** Serena Fu, Nicole Huang, Yiing-Jenq Chou

**Affiliations:** Author Affiliations: Serena Fu, Institute of Public Health, School of Medicine, National Yang Ming University, Taipei, Taiwan; Nicole Huang, Institute of Public Health, School of Medicine, National Yang Ming University, and Taipei City Hospital, Taipei, Taiwan.

## Abstract

**Introduction:**

Chronic conditions are the leading causes of death and disability worldwide. Providing care to people diagnosed with a chronic disease is challenging, and controlling multiple chronic conditions (MCCs) can be overwhelming, particularly in rapidly aging societies. The objective of this study was to examine the prevalence of MCCs from 2000 to 2010 in Taiwan.

**Methods:**

A random sample of 1 million representative National Health Insurance beneficiaries in 3 years (2000, 2005, and 2010) was obtained from the Taiwan National Health Insurance Research Database to examine the prevalence of MCCs. Chronic Condition Indicator and Clinical Classifications Software were used to determine and classify codes from the *International Classification of Diseases, Ninth Revision*. People who had 2 or more conditions among the 15 categories of conditions were defined as having MCCs.

**Results:**

The prevalence of MCCs increased from 9.6% in 2000 to 17.1% in 2010. The highest prevalence of MCCs was found among people aged 65 years or older (42.3% in 2000 and 64.5% in 2010, a relative increase of 52.5%). However, the highest rate of increase was found among people younger than 18 years (0.5% in 2000 and 1.6% in 2010, a relative increase of 220.0%).

**Conclusion:**

MCCs are increasingly prevalent among the older (≥65 y) population and among children and adolescents. Prevention and early intervention programs targeted to certain age groups may be required. If the increase in MCCs continues rapidly, the management of people diagnosed with MCCs would challenge the capacity of the health care system in Taiwan.

## Introduction

Chronic conditions have become a major challenge to health care systems in the 21st century (1,2). The challenge is especially great among older people (those aged 65 or older); as people age, they become more susceptible to developing chronic conditions (3). Therefore, as the size of the older population increases, the prevalence of chronic conditions increases. Multiple chronic conditions (MCCs) are associated with poor health outcomes, such as unnecessary hospitalizations, duplicate treatments, conflicting instructions, functioning disability, and mortality (1,2,4,5).

Providing appropriate care to people diagnosed with a chronic disease is challenging, and controlling MCCs can be overwhelming for patients, families, providers, and society (6). People diagnosed with MCCs may have more complex clinical needs and more often receive health care services from multiple providers than people diagnosed with 1 chronic condition (3). Development of integrated health care services has emerged as an essential issue and a research priority to ensure the health of people diagnosed with MCCs and the efficient use of health care resources (5,7–9).

A first step in confronting these challenges is to provide insights into the scope of the population with MCCs (10). Several studies examined the prevalence of MCCs in Australia, Canada, Europe, and the United States (10–15). A recent review summarizing 33 population-based studies on multimorbidity in Australia, Canada, the Netherlands, Spain, Sweden, and the United States found a prevalence of MCCs ranging from 20% to 30% (1). Although much is known about the prevalence of MCCs, well-designed studies on the trends in prevalence of MCCs are scarce.

Reports on the prevalence of MCCs are based primarily on cross-sectional studies and restricted to the older population, which may be problematic for generalizing findings to the overall population. Evidence is mounting that although older people are at an especially high risk of MCCs, MCCs are also common among other age groups. Although the prevalence of MCCs increases as age increases, the absolute number of people diagnosed with MCCs is high in people younger than 65 (12,16,17). One of the most critical decisions in studying MCCs is the selection of conditions to be assessed. The selection of 12 or more conditions may result in data that do not show much variation (18). On the other hand, focusing on a small number of conditions may underestimate the true burden of MCCs. Diabetes, cardiovascular disease, hypertension, cancer, respiratory disease, joint disorders, and heart disease are the conditions most commonly studied (19). Conditions commonly found among younger people, such as eye disorders and digestive diseases, are less likely to be studied. Data collected over time on the prevalence of MCCs among the general population and all age groups can show the temporal dynamics of MCCs and improve our understanding of the magnitude of the burden.

A rapid increase in the percentage of older people and dramatic lifestyle changes in several Asian countries leave these countries with a short time to cope with the challenges of providing care to people diagnosed with MCCs. Taiwan is an illustrative example: it has one of the fastest-growing older populations in the world. Taiwan’s older population might grow faster than European and North American countries and surpass Japan to become the world’s “most aged” country by 2033 (20,21). More empirical evidence on changes in epidemiological patterns and MCCs is required to re-engineer the health care system in Taiwan, which was originally designed to handle acute and episodic diseases.

The objective of this study was to investigate trends in the prevalence of MCCs in Taiwan using nationally representative cohorts of National Health Insurance (NHI) beneficiaries. 

## Methods

This study was a descriptive cross-sectional analysis of prevalence estimates over time among age and sex categories in an Asian population. Taiwan implemented the NHI program in March 1995; this program offers comprehensive health insurance coverage to all residents. The NHI program covered approximately 96% of the population of 23.76 million by 2000, and today, more than 99% of the population is covered. The NHI maintains the National Health Insurance Research Database, which contains enrollment and claims data on use of inpatient, outpatient, and emergency services and prescription drugs. For research purposes every 5 years, the National Health Research Institutes randomly samples cohorts of 1 million NHI beneficiaries representative of the general Taiwanese population. For this study, we obtained data sets of 3 random samples of 1 million insurance beneficiaries in 3 years: 2000, 2005, and 2010. We excluded records for beneficiaries that lacked information on age, sex, socioeconomic status (SES), or region of residence. We found no significant difference in age or sex between the samples and all NHI beneficiaries (22).

We used the Chronic Condition Indicator (23) to determine *International Classification of Diseases, Ninth Revision* (ICD-9) diagnosis codes in the administrative data and to categorize ICD-9 diagnosis codes into 1 of 2 categories: chronic and nonchronic. Clinical Classifications Software (CCS) (24) was used to classify codes that indicated similar clinical characteristics into several clinically meaningful categories (Appendix). Beneficiaries who had at least 1 inpatient claim for a chronic condition and those who made at least 3 outpatient visits classified with the same CCS code for a chronic condition within 1 year were defined as patients that had a chronic condition.

There is no standard definition for MCCs. Many studies define MCCs as 2 or more conditions, and some define MCCs as 3 or more conditions. The use of 3 or more conditions may be more meaningful for clinicians than a count of 2 or more when the study population is older (18,25). Because our objective was to examine trends in the prevalence of MCCs in the general population, we chose to adopt the definition of 2 or more conditions. We also chose to study the 15 most prevalent chronic conditions among the study population (hepatitis, cancer, diabetes, hyperlipidemia, gout and other crystal arthropathies, depression, eye disorders, nervous system disorders, hypertension, heart disease, cerebrovascular disease, respiratory disease, digestive disease, genitourinary disease, and joint disorder) to reflect the overall burden and to present a comprehensive picture among all age groups. Our approach is the approach most commonly used in various relevant studies (1,26). The 15 chronic conditions selected are commonly selected in studies conducted in Taiwan (27,28). We also determined the 3 most common types of dyads and triads by age groups; these dyads and triads are not mutually exclusive (ie, an individual could be in more than 1 dyad or triad).

Information on age, sex, SES, and region of residence was obtained from the enrollment files. Age was classified into 5 categories: younger than 18 years, 18 to 24 years, 25 to 44 years, 45 to 64 years, and 65 years or older. SES was constructed on the basis of the beneficiary’s insurable wages and occupation. NHI enrollment is mainly carried out through payroll deduction for people with well-defined monthly wages and through tax payments for farmers, fishermen, and others not having a well-defined monthly wage. SES for people with well-defined monthly wages was divided into 3 categories: less than NT$ (Taiwanese new dollar) 20,000 (US$667), NT$20,000 (US$667) to NT$39,999 (US$1,333), and more than NT$39,999 (US$1,333) per month. Those without a well-defined monthly wage were farmers and fishermen, local office beneficiaries, veterans; and low-income people; they were categorized as “fixed amount.” Dependents were classified in the same category as the primary insured person. Beneficiaries were classified according to geographical region into 1 of 4 regions: northern, central, southern, and eastern. We determined the distribution of demographic characteristics for each sample according to the number of chronic conditions. To ascertain cross-sectional prevalence over time in the general population, we calculated the prevalence for each study year by dividing the number of beneficiaries diagnosed with MCCs by the total number of beneficiaries for each year. The relative change in overall MCC prevalence between 2000 and 2010 was calculated by dividing the difference between the estimate for 2000 and 2010 by the estimate for 2000 and expressed as a percentage. Age- and sex-specific prevalence was also estimated. To assess trends in prevalence rates during the study period, we used the χ^2^ test and when appropriate, the ptrend command in Stata (StataCorp LP). Statistical analyses were conducted using Stata version 10.0 and SAS version 9.2 (SAS Institute Inc)*.* A *P* value less than .05 was considered significant.

## Results

The final samples were 999,635 in 2000, 999,974 in 2005, and 999,998 in 2010 (Table 1). The mean age was 34.8 years in 2000, 36.4 years in 2005, and 38.7 years in 2010. The percentage of women increased from 48.6% in 2000 to 50.8% in 2010. The overall prevalence of MCCs increased from 9.6% in 2000 to 13.5% in 2005 and 17.1% in 2010, a relative increase of 78.1%. The prevalence of MCCs in 4 categories (2 conditions, 3 conditions, 4 conditions, or ≥5 conditions) also increased from 2000 to 2010 (Figure). The relative increase in prevalence from 2000 to 2010 ranged from 57.4% (for people with 2 conditions) to 121.0% (for people with ≥5 conditions).

**Table 1:** Selected Demographic Characteristics of Samples, by Number of Chronic Conditions in 2000, 2005, and 2010, Taiwan^a^^,^ b

Characteristic	2000 (N = 999,635)			2005 (N = 999,974)			2010 (N = 999,998)			*P* Value^c^
	No. of Chronic Conditions			No. of Chronic Conditions			No. of Chronic Conditions			
	1	2–5	>5	1	2–5	>5	1	2–5	>5	
**No. of people**	118,118	91,924	4,062	150,196	128,256	7,134	158,351	161,139	9,658	**—**
**Overall prevalence**	11.8	9.2	0.4	15.0	12.8	0.7	15.8	16.1	1.0	<.001
**Sex**										
Female	14.0	10.0	0.4	17.1	13.3	0.7	18.0	16.5	0.9	<.001
Male	9.7	8.4	0.4	12.9	12.4	0.8	13.6	15.7	1.1	<.001
**Mean age, y**	40.4	60.3	69.8	39.3	60.3	71.1	40.1	60.8	71.7	—
**Age group**										
<18 y	8.3	0.5	0.0	13.0	1.1	0.0	15.5	1.6	0.0	<.001
18–24 y	8.0	0.9	0.0	11.3	1.5	0.0	11.3	1.6	0.0	<.001
25–44 y	10.8	3.2	0.0	14.0	4.7	0.0	14.9	5.6	0.0	<.001
45–64 y	17.8	19.0	0.5	18.8	22.0	0.7	18.8	25.5	0.9	<.001
≥65 y	16.0	39.4	2.9	17.4	52.6	5.1	15.6	58.4	6.1	<.001
**Socioeconomic status^d^**										
<$20,000	10.5	6.1	0.2	14.1	8.1	0.3	14.2	7.7	0.3	<.001
$20,000–$39,999	12.0	7.0	0.2	15.4	10.5	0.4	16.6	13.2	0.6	<.001
≥40,000	13.3	9.1	0.3	15.9	12.0	0.5	17.2	15.7	0.7	<.001
Fixed	12.4	14.6	0.8	15.0	19.4	1.5	14.7	23.9	1.9	<.001
**Region**										
Northern	11.5	8.6	0.4	14.5	11.9	0.6	15.4	15.1	0.9	<.001
Central	11.8	9.1	0.4	15.6	12.7	0.6	16.5	16.0	0.9	<.001
Southern	12.3	10.2	0.5	15.7	14.4	0.9	16.3	17.8	1.1	<.001
Eastern	11.9	11.0	0.4	14.6	15.6	0.8	15.2	20.0	1.3	<.001

Abbreviations: N, number; SES, socioeconomic status.

a Three sets of random samples of National Health Insurance beneficiaries were obtained from the Taiwan National Health Insurance Research Database (22).

b All values are percentages unless otherwise indicated.

c The trend for 2 to 5 chronic conditions was tested by χ^2^.

d Socioeconomic status was constructed on the basis of the beneficiary’s insurable wages and occupation. For people with well-defined monthly wages, it was divided into 3 categories: less than NT$ (Taiwanese new dollar) 20,000, NT$20,000 to NT$39,999, and more than NT$39,999. Those without a well-defined monthly wage were farmers and fishermen, local office beneficiaries, veterans; and low-income people; they were categorized as “fixed amount.”

**Figure:**
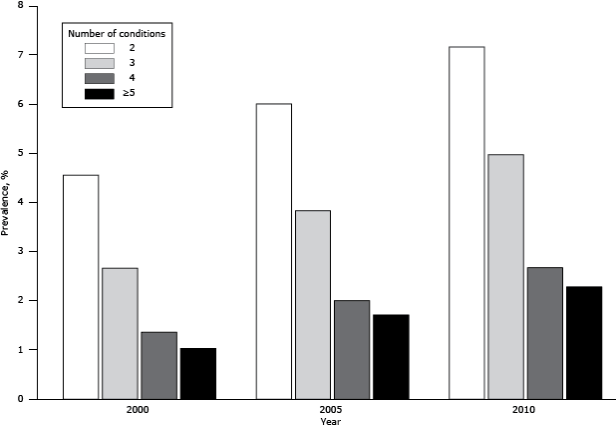
Prevalence of multiple chronic conditions, by number of chronic conditions among the general population in Taiwan in 2000, 2005, 2010. Source of data: Taiwan National Health Insurance Research Database (22). **Table:** 

No. of Conditions	2000	2005	2010
**2**	4.55	6.00	7.16
**3**	2.66	3.83	4.97
**4**	1.36	2.00	2.67
**≥5**	1.03	1.71	2.28

The prevalence of MCCs was higher among females than among males in each study year. The overall prevalence of MCCs increased more among males (by 90.9%) than among females (by 67.3%) from 2000 to 2010. As expected, whereas 8.8% in 2000, 14.1% in 2005, and 17.1% in 2010 of those aged younger than 18 had at least 1 chronic condition, the prevalence was highest in those aged 65 or older (58.3% in 2000, 75.1% in 2005, and 80.1% in 2010). The greatest absolute increase in prevalence of MCCs was found among those aged 65 or older (from 42.3% to 64.5%), but the greatest rate of increase was found among those aged younger than 18 (from 0.5% to 1.6%, a relative increase of 220.0%), followed by the rate of increase for these age groups: 18 to 24 (by 77.8%), 25 to 44 (by 75.0%), 65 or older (by 52.5%), and 45 to 64 (by 35.4%). By income group, the prevalence of MCCs was lowest in the low-income group; the highest relative increase was found in the middle-income group. We found the largest relative and largest absolute increase in prevalence of MCCs among people residing in the eastern region.

The prevalence of MCCs varied according to age and sex (Table 2). We found a significant difference in the relative percentage change from 2000 to 2010 in the prevalence of MCCs among men (64.6%) and women (13.8%) aged 45 to 64. Whereas the prevalence of MCCs among men aged 45 to 64 increased significantly from 16.1% in 2000 to 26.5% in 2010, the prevalence among women aged 45 to 64 women was stable. By contrast, the prevalence of MCCs was similar for men and women aged 65 or older during the study period. The prevalence of MCCs among women aged 65 or older increased from 43.9% in 2000 to 65.9% in 2010, whereas the prevalence among men in this age group from 40.8% in 2000 to 63.1% in 2010.

**Table 2:** Age- and Sex-Specific Prevalence of Multiple Chronic Conditions in 2000, 2005, and 2010, Taiwan^a^

Age and Sex	2000		2005		2010		Relative Change From 2000 to 2010, %	*P* for Trend
	Sample, n	Prevalence, n (%)	Sample, n	Prevalence, n (%)	Sample, n	Prevalence, n (%)		
**Female**								
<18 y	109,698	484 (0.4)	101,358	976 (1.0)	86,668	1,236 (1.4)	223.2	<.001
18–24 y	58,289	650 (1.1)	53,950	918 (1.7)	47,795	989 (2.1)	85.6	<.001
25–44 y	171,931	5,836 (3.4)	175,831	8,134 (4.6)	173,037	9,633 (5.6)	64.0	<.001
45–64 y	97,768	22,496 (23.0)	119,202	28,490 (23.9)	139,687	36,588 (26.2)	13.8	<.001
≥65 y	48,286	21,173 (43.9)	53,829	31,660 (58.8)	60,388	39,780 (65.9)	50.2	<.001
All	485,972	50,639 (10.4)	504,170	70,178 (13.9)	507,575	88,226 (17.4)	66.8	<.001
**Male**								
<18 y	119,158	714 (0.6)	110,855	1,327 (1.2)	95,091	1,585 (1.7)	178.2	<.001
18–24 y	61,327	386 (0.6)	47,296	574 (1.2)	45,007	493 (1.1)	74.0	<.001
25–44 y	178,396	5,572 (3.1)	166,698	7,980 (4.8)	159,359	8,981 (5.6)	80.4	<.001
45–64 y	99,120	15,944 (16.1)	117,483	25,098 (21.4)	137,195	36,316 (26.5)	64.6	<.001
≥65 y	55,662	22,731 (40.8)	53,472	30,233 (56.5)	55,771	35,196 (63.1)	54.5	<.001
All	513,663	45,347 (8.8)	495,804	65,212 (13.2)	492,423	82,571 (16.8)	89.9	<.001

a Three sets of random samples of National Health Insurance beneficiaries were obtained from the Taiwan National Health Insurance Research Database (22).

Among children and adolescents with 2 or more MCCs, the most prevalent dyad was eye disorder/respiratory disease, followed by respiratory disease/genitourinary disease, which was also the most prevalent dyad for those aged 18 to 24 (Table 3); these combinations increased in prevalence during the study period. The most common co-occurring condition (in dyads) was genitourinary disease for those aged 25 to 44 and hypertension for those aged 45 or older. Among children and adolescents who had at least 3 chronic conditions, eye disorder/respiratory disease/genitourinary disease was the leading combination in 2005, and its prevalence increased in 2010. This triad was also one of the most prevalent among those aged 18 to 24, and its prevalence also increased during the study period. A common triad found among those aged 25 to 64 was diabetes/hyperlipidemia/hypertension; this triad more than doubled in prevalence from 2000 to 2010. Diabetes/hypertension/heart disease was prevalent among those aged 45 or older, whereas hypertension/heart disease/joint disorder was the most prevalent among those 65 or older, and its prevalence was stable during the study period.

**Table 3:** Most Prevalent Dyads of Chronic Conditions Among People With 2 or More Chronic Conditions and Most Prevalent Triads Among People With 3 or More Chronic Conditions, by Age Group in 2000, 2005, and 2010, Taiwan^a^^,^ b

Age Group	2000		2005		2010	
**Dyads**						
**<18 y**	Eye/Resp	667 (55.7)	Eye/Resp	1,434 (62.3)	Eye/Resp	1,783 (63.2)
	Resp/Genito	114 (9.5)	Resp/Genito	233 (10.1)	Resp/Genito	271 (9.6)
	Resp/Digest	102 (8.5)	Resp/Digest	191 (8.3)	Resp/Digest	209 (7.4)
**18–24 y**	Resp/Genito	137 (13.2)	Resp/Genito	288 (19.3)	Resp/Genito	336 (22.7)
	Eye/Genito	94 (9.1)	Eye/Genito	142 (9.5)	Eye/Resp	158 (10.7)
	Eye/Resp	71 (6.9)	Eye/Resp	116 (7.8)	Eye/Genito	129 (8.7)
**25–44 y**	Hepa/Digest	836 (7.3)	Resp/Genito	1,388 (8.6)	Resp/Genito	1,837 (9.9)
	Depress/Digest	803 (7.0)	HLD/HTN	1,088 (6.8)	HLD/HTN	1,747 (9.4)
	Resp/Genito	704 (6.2)	Hepa/Digest	1,022 (6.3)	Diabetes/HLD	1,508 (8.1)
**45–64 y**	Genito/Joint	5,739 (14.9)	Diabetes/HTN	8,842 (16.5)	HLD/HTN	16,130 (22.1)
	Diabetes/HTN	5,006 (13.0)	HLD/HTN	8,504 (15.9)	Diabetes/HTN	13,962 (19.2)
	HTN/Heart	4,911 (12.8)	HTN/Heart	7,301 (13.6)	Diabetes/HLD	11,900 (16.3)
**≥65 y**	HTN/Heart	10,632 (24.2)	HTN/Heart	15,400 (24.9)	Diabetes/HTN	18,521 (24.7)
	HTN/Joint	8,260 (18.8)	HTN/Joint	13,127 (21.2)	HTN/Heart	18,334 (24.5)
	Diabetes/ HTN	7,800 (17.8)	Diabetes/HTN	13,084 (21.1)	HTN/Joint	16,678 (22.2)
**Triads**						
**<18 y**	Depress/Resp/Digest	3 (6.5)	Eye/Resp/Genito	20 (17.7)	Eye/Resp/Genito	29 (20.9)
	Depress/Digest/Genito	3 (6.5)	Eye/Resp/Digest	17 (15.0)	Eye/Resp/Digest	17 (12.2)
	Heart/Resp/Digest	3 (6.5)	Resp/Digest/Genito	8 (7.1)	Depress/Eye/Resp	13 (9.4)
**18–24 y**	Depress/Resp/Digest	8 (6.4)	Depress/Resp/Genito	18 (8.2)	Eye/Resp/Genito	20 (9.5)
	Eye/Resp/Genito	8 (6.4)	Eye/Resp/Genito	17 (7.7)	Resp/Digest/Genito	14 (6.6)
	Hepa/HLD/Gout	7 (5.6)	Hepa/HLD/Gout	12 (5.5)	Depress/Resp/Digest	12 (5.7)
**25–44 y**	Diabetes/HLD/HTN	123 (3.7)	Diabetes/HLD/HTN	272 (5.6)	Diabetes/HLD/HTN	562 (9.6)
	Depress/HTN/Heart	114 (3.4)	HLD/Gout/HTN	190 (3.9)	HLD/Gout/HTN	263 (4.5)
	Hepa/Depress/Digest	113 (3.4)	HLD/HTN/Heart	187 (3.9)	HLD/HTN/Heart	221 (3.8)
**45–64 y**	HTN/Genito/Joint	1,274 (6.8)	Diabetes/HLD/HTN	3,344 (12.1)	Diabetes/HLD/HTN	6,619 (16.7)
	Diabetes/HLD/HTN	1,220 (6.6)	HLD/HTN/Heart	1,963 (7.1)	HLD/HTN/Heart	3,240 (8.2)
	Diabetes/HTN/Heart	1,078 (5.8)	Diabetes/HTN/Heart	1,761 (6.4)	Diabetes/HTN/Heart	2,394 (6.0)
**≥65 y**	HTN/Heart/Joint	3,039 (10.7)	HTN/Heart/Joint	4,642 (10.9)	Diabetes/HLD/HTN	6,488 (12.1)
	HTN/Heart/Resp	2,641 (9.3)	Diabetes/HTN/Heart	4,247 (10.0)	HTN/Heart/Joint	5,704 (10.7)
	Diabetes/HTN/Heart	2,574 (9.1)	Eye/HTN/Heart	3,934 (9.2)	Diabetes/HTN/Heart	5,584 (10.5)

Abbreviations: Eye, eye disorder; Resp, respiratory disease; Genito, genitourinary disease; Digest, digestive disease; Hepa, hepatitis; Depress, depression; HLD, hyperlipidemia; HTN, hypertension; Joint, joint disorder; Heart, heart disease; Gout, gout and other crystal arthropathies.

a All values are number (percentage).

b Three sets of random samples of National Health Insurance beneficiaries were obtained from the Taiwan National Health Insurance Research Database (22).

## Discussion

This study is one of the first population-based investigations of trends in the prevalence of MCCs among all age groups of a general population in Asia from 2000 to 2010. Based on the nationally representative samples of nearly 1 million beneficiaries in 2000, 2005, and 2010, the prevalence of 2 or more chronic conditions increased from 9.6% in 2000, to 13.5% in 2005 and 17.1% in 2010. Several factors may have contributed to the rise in MCCs, including the natural outcomes of the aging process and an increase in longevity among the Taiwanese. As people live longer, their likelihood of developing a chronic condition increases, and the duration of disease lengthens. The rise in the estimates of MCCs may also result from earlier and better detection of disease — which may be caused by health care promotion activities and an emerging public awareness of chronic conditions.

Most clinical guidelines for chronic diseases are principally designed to treat diseases as if they occur in isolation. This study further determined that the prevalence of MCCs surpassed the prevalence of single chronic conditions during the study period. This finding illustrates the importance of MCCs in developing treatment guidelines and reforming current clinical practices and health care systems.

In this study, estimates of the prevalence of MCCs for each age group were lower than estimates reported for Western populations. This difference may be explained by differences in ethnicity and lifestyle between Asian and Western populations (29). It is well established that a strong association exists between age and MCCs. Our study shows that the prevalence of at least 1 chronic condition among those aged younger than 18 increased from 8.8% to 17.1% from 2000 to 2010 and that more than half of people who have MCCs in Taiwan were younger than 65. These findings indicate that Taiwan must respond soon to the challenge of a rapid increase in the prevalence of MCCs.

Consistent with findings from other studies, our findings indicate that the prevalence of MCCs among women is higher than the prevalence among men, regardless of age (2,13). The “survivor effect” may provide 1 explanation for the sex difference: the longer life expectancy among women may put them at higher risk of exposure to nonfatal chronic conditions. Sex differences in behaviors of seeking health care may also explain sex differences in the prevalence of MCCs. For example, women may be more likely than men to use ambulatory care services; thus, their chronic conditions may be detected more easily than those of men (1,30).

This study has several strengths. One, we used a nationally representative sample and a large sample size, which enabled us to generalize our findings and assess the prevalence of MCCs among various age groups. Two, the NHI database allowed us to observe prevalence over time. Three, we used a small number but a large spectrum of chronic conditions recommended for measuring the prevalence of MCCs (18). The prevalence of MCCs reported here was based on a list of chronic conditions that included the most prevalent conditions in Taiwan; thus, our findings are representative of the actual prevalence among the general population. Four, our study gives a first insight into the trends in the most common types of dyads and triads of chronic conditions by age group; understanding disease patterns over time for each age group is critical. Five, our study not only highlights the disease combinations that require the greatest health care but also serves as a direction for the future design of best practice guidelines.

Our study has several limitations. One, by counting the number of chronic conditions, we scored all conditions equally and did not consider the severity of any condition. Two, our study was based exclusively on administrative claims data; a limitation of claims data is the potential unreliability of the information on diagnoses, which may lead to misclassification and intentional or unintentional bias. We attempted to reduce such bias by adding strict criteria to the definition of chronic conditions, and to minimize misclassification, we included only patients who had at least 1 inpatient claim and patients who had 3 outpatient diagnoses. Thus, the prevalence of MCCs measured in our study may be underestimated.

Despite these limitations, our study contributes to the literature by presenting a comprehensive picture of the prevalence of MCCs during 10 years. The aging of the baby-boomer population is expected to cause a large burden of MCCs. Several Asian countries, including Taiwan, provide examples of such rapidly aging baby-boomer populations. As greater numbers of older people live longer, and as lifestyles continue to modernize, a rise in the prevalence of MCCs is foreseen. We also foresee a rise among the younger population. The increasing prevalence of MCCs challenges the health care system to provide timely and appropriate care. Knowledge about disease patterns can help optimize preventive strategies and early recognition of concurrent conditions. Further research is required to improve understanding of MCCs and organize health care systems for people who have them.

## Appendix. Selection of 15 Chronic Conditions by Clinical Classification Software (CCS) Code

**Table:**

Chronic Condition	CCS code
Hepatitis	1.3
Cancer	2.1, 2.2, 2.3, 2.4, 2.5, 2.6, 2.7, 2.8, 2.9, 2.10, 2.11, 2.12, 2.13, 2.14, 2.15, 2.16
Diabetes	3.2, 3.3
Hyperlipidemia	3.6
Gout and other crystal arthropathies	3.7
Depression	5.2, 5.8
Eye disorder	6.7
Nervous system disorders	6.9
Hypertension	7.1
Heart disease	7.2
Cerebrovascular disease	7.3
Respiratory disease	8.1, 8.2, 8.3, 8.4, 8.5, 8.6, 8.7, 8.8, 8.9
Digestive disease	9.1, 9.2, 9.3, 9.4, 9.5, 9.6, 9.7, 9.8, 9.9, 9.10, 9.11, 9.12
Genitourinary disease	10.1, 10.2, 10.3
Joint disorder	13.2, 13.3, 13.4
